# Evaluating YouTube videos on sialendoscopy as an educational resource for patients

**DOI:** 10.1002/lio2.991

**Published:** 2022-12-16

**Authors:** Daniel J. Campbell, Kevin Xiao, Eric Mastrolonardo, David M. Cognetti

**Affiliations:** ^1^ Department of Otolaryngology – Head and Neck Surgery Thomas Jefferson University Philadelphia Pennsylvania USA; ^2^ Department of Otolaryngology – Head and Neck Surgery University of Missouri Columbia Missouri USA

**Keywords:** minimally invasive surgery, salivary stones, sialadenitis, sialendoscopy, YouTube

## Abstract

**Objectives:**

Patients are increasingly relying on YouTube for health information. We objectively evaluated the quality and comprehensiveness of sialendoscopy YouTube videos available to patients. We further investigated the relationship between video content and video popularity.

**Methods:**

We identified 150 videos using the search term “sialendoscopy.” Videos were excluded if they were lectures for medical professionals, operating room (OR) recordings, unrelated, non‐English, or non‐audio. Video quality and comprehensiveness were evaluated using modified DISCERN criterion (range: 5–25) and novel sialendoscopy criterion (NSC, range: 0–7), respectively. Secondary outcomes included standard video metrics and Video Power Index to measure popularity. Videos were classified binarily by uploader type as from an academic medical center or from other sources.

**Results:**

Twenty‐two (14.7%) of 150 videos were included for review, with 7 (31.8%) uploaded from academic medical institutions. One hundred‐nine (72.7%) videos were excluded as lectures for medical professionals or OR recordings. Overall mean modified DISCERN (13.45 ± 3.42) and NSC (3.05 ± 0.96) scores were low; however, videos uploaded by academic medical institutions were significantly more comprehensive (NSC mean difference = 0.98, 95% CI: 0.16–1.80, *p* = .02). There were no significant correlations between video popularity and objective measures of quality or comprehensiveness.

**Conclusions:**

This study highlights the paucity and low quality of sialendoscopy videos for patients. More popular videos are not higher quality, and most videos are targeted more toward physicians rather than patients. As YouTube becomes increasingly used by patients, there is opportunity for otolaryngologists to produce more informative videos for patients while implementing targeted strategies to increase viewership.

**Level of Evidence:**

NA.

## Graphical abstract

Patients are increasingly watching YouTube videos as a means of educating themselves on their potential treatment options. YouTube videos available to patients on sialendoscopy are overall sparse and of low quality. Furthermore, more popular videos are not of higher quality.

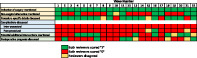

## 1 INTRODUCTION

Patients are increasingly using online resources for health information. Recent data suggest that about 80% of Internet users look for health information online, and of these users, 56% look specifically for information on medical treatments or procedures.^1^ Although numerous websites host video content, YouTube is by far the largest and most‐visited repository of videos worldwide.^2^ The most recent 2020 Health Information National Trends Survey estimates that 39.7% of Americans watched health‐related videos on YouTube, making YouTube the most‐accessed video streaming website specifically for health information.^2^, ^3^ As of August 2021, YouTube's mobile application is the most‐used smartphone application in the United States, and its user base is projected to continue growing as consumers increasingly access information from mobile devices.^4^

Sialendoscopy is a diagnostic and therapeutic tool used to manage sialolithiasis and sialadenitis refractory to first‐line, conservative treatment. It is a minimally invasive technique that has been shown to preserve function and spare excision of salivary glands in non‐neoplastic obstructive pathologies.^5^, ^6^ Sialendoscopy is highly effective in relieving salivary obstruction, with reported success rates approaching 91% and is associated with minimal complications.^7^, ^8^ Since its introduction in the 1990s, usage of sialendoscopy has increased, and it is now performed in a variety of surgical settings.^9^, ^10^, ^11^, ^12^

Despite the growth in patient use of YouTube and clinical use of sialendoscopy, the quality of the information found in sialendoscopy YouTube videos has not been investigated. Given that YouTube videos are unregulated and often contain incomplete or inaccurate health information, there is growing concern over the quality of information that patients access on the website.^13^, ^14^, ^15^ Thus, this study seeks to objectively evaluate the quality and comprehensives of YouTube videos as a source of health information for patients on sialendoscopy. We hypothesize that the information within YouTube videos available for patient on sialendoscopy is of low quality and comprehensiveness.

## 2 MATERIALS AND METHODS

This research study received exemption from the Institutional Review Board at Thomas Jefferson University. Videos were searched using the Google Chrome web browser. To minimize user search result bias, browsing history, cookies, and cache were cleared before searching, and all videos were accessed via Google Chrome's private browsing feature. On March 26, 2021, “www.youtube.com” was searched using default settings for the term “sialendoscopy.”

The first 150 videos were identified. A search of 150 videos is likely far beyond what a typical user would do, allowing for analysis of the entire breadth of sialendoscopy video content and proper powering of the study.^16^, ^17^ Videos were excluded if they were (1) lecture format videos intended for medical professionals, (2) non‐English videos, (3) duplicates, (4) surgical and/or operating room (OR) technique‐specific videos, (5) irrelevant videos (defined as videos with titles that do not mention sialendoscopy and contain less than 50% of video time spent discussing sialendoscopy or topics directly related to sialendoscopy), or (6) videos without both audio and subtitles.

Videos were classified based on their uploader as either from an academic medical institution (“academic”) or from any other uploader source (“other”). Other uploaders included private medical institutions, news and media agencies, and individual users. This video stratification is consistent with previous literature^18^, ^19^, ^20^ and allowed for proper powering of the study.

Primary outcome measures were scores from two video scoring criteria: a novel sialendoscopy criteria (NSC) score and a modified DISCERN score. Each video was independently scored by two authors. The authors created the NSC to evaluate the comprehensiveness of sialendoscopy information within a video. It was designed with input from other novel scoring criteria in the literature, and it represents the breadth of information an otolaryngologist would likely present to patients undergoing sialendoscopy.^21^, ^22^, ^23^ Scoring (range: 0–7) is based on the discussion of information pertaining to surgical indications, alternatives to surgery, complications, potential need for additional intraoperative interventions, and postoperative prognosis (Table 1).

**TABLE 1:** Novel sialendoscopy criteria scoring system

Score range: 0–7 points
Surgical indications (1 pt)
Non‐surgical alternatives (1 pt)
Procedure‐specific details (1 pt)
Complications (2 pts) Intraprocedural (1 pt) Postprocedural (1 pt)
Potential need for additional intraoperative intervention (1 pt)
Postoperative prognosis (1 pt)

*Note*: Novel sialendoscopy criteria (NSC) used to assess for comprehensiveness of information in videos. Each item is scored 0 or 1 and combined for a total score ranging from 0 to 7. Videos received a “1” if the corresponding criterion was adequately discussed within the video body.

The DISCERN criteria are a validated scoring system developed to help health consumers judge information about treatment choices (i.e., sialendoscopy), and it has been previously used in the literature to evaluate the overall quality and reliability of health information in YouTube videos.^21^, ^22^, ^24^, ^25^, ^26^ The original DISCERN instrument is a 15‐item survey broken up into two sections: reliability (items 1–8) and specific details about treatment choices (items 9–15).^24^ The modified DISCERN implements and combines the reliability items (1–8) into a 5‐item score, each graded on a scale of 1–5, yielding a total score of 5–25 (Table 2).^21^ Items 4 and 5 from the original DISCERN instrument are excluded, as they are specific to written forms of patient education. Items 9–15 are excluded from the modified DISCERN because they (1) are addressed more specifically in the context of sialendoscopy within the NSC, or (2) assume multiple treatment modalities are being compared, whereas our investigation evaluates a single treatment. The DISCERN bias score was the third criteria in the modified DISCERN score and was used to assess the presence of bias in videos (Table 2).

**TABLE 2:** Modified DISCERN criteria scoring system^a^

Question	Scoring: Range 5–25
1. Are the aims clear and achieved?	1–5
2. Are reliable sources of information used (i.e., publication cited, speaker is otolaryngologist)?	1–5
3. Is the information balanced and unbiased?	1–5
4. Are additional sources of information listed for patient reference?	1–5
5. Are areas of uncertainty mentioned?	1–5

*Note*: Modified DISCERN criteria used to evaluate the quality of information in videos. Each item is scored on a scale of 1 to 5 and combined for a total score ranging from 5 to 25.

^a^ Adapted from Wu et al.^19^

Secondary outcome measures included: (1) audiovisual (AV) quality score, (2) reported video metrics, and (3) calculated video metrics. The AV quality score has previously been used to evaluate YouTube videos and ranges from zero to three, with higher scores indicating clearer, more professional editing (Table 3).^16^ Reported video metrics included the number of views, comments, likes, dislikes, days since upload, and duration. Calculated video metrics included like ratio (likes/[likes + dislikes]), view ratio (views/days since upload), and video power index (VPI). VPI is a previously published measure of video popularity calculated as: VPI = ([view ratio * like ratio]/100).^19^, ^21^, ^22^, ^27^, ^28^ Data used for basic and calculated video metrics were collected at the date of the initial YouTube search (March 26, 2021).

**TABLE 3:** Audiovisual quality scoring system^a^

Score Range: 0–3 points
*3—Excellent*: clear, professional editing
*2—Average*: non‐professional editing
*1—Poor*: blurry, out of focus, unintelligible
*0—Unable to view*

*Note*: Assessment of video audiovisual quality.

^a^ Adapted from Singh et al.^20^

All statistical analyses were performed using IBM SPSS Statistics Version 27. Statistical significance was set at *p* < .05. Intraclass Correlation Coefficient (ICC) was calculated based on NSC and modified DISCERN scores from both reviewers, with ICC > 0.90 defined as “excellent” reliability.^29^ Basic descriptive statistics and Pearson Correlation Coefficient were calculated for all primary outcomes (using the mean of both reviewers' scores) and secondary outcomes, regardless of uploader type. Primary and secondary outcomes were compared between uploader types (academic and other) using an independent *t*‐test supplemented by Levene's test and Cohen's *D* measurement.

## 3 RESULTS

Of the 150 videos identified from the initial YouTube search, 22 videos (14.7%) met inclusion criteria. The 128 excluded videos included the following: 8 lecture‐format videos intended for a live audience, 9 non‐English videos, 101 surgical or OR technique‐specific videos, 7 irrelevant videos, and 3 videos without both audio and subtitles. Of the 22 included videos, 7 (31.8%) were uploaded from an academic medical institution, and 15 (68.2%) were uploaded from other sources. Other uploader sources included 10 videos from private medical institutions, 4 videos from independent users, and 1 video from a news/media organization (Table 4).

**TABLE 4:** Videos stratified by uploader source

Uploader source	Number of videos (% of total videos)
*Academic Medical Institution*	7 (31.8%)
*Other Uploader Sources*	15 (68.2%)
Private medical institution	10 (45.5%)
Independent uploader	4 (18.2%)
News/media organization	1 (4.5%)

*Note*: Videos included in the study stratified by uploader source. Other uploaders included all videos that were not from academic medical institutions and could be categorized into three categories: private medical institutions, independent uploaders, and news/media organizations.

**FIGURE 1:**
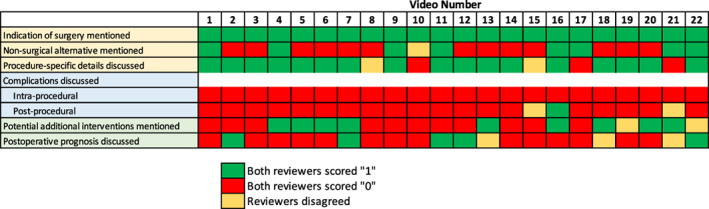
Reviewer scoring for novel sialendoscopy criteria (NSC) by two authors. A score of “1” was given if a reviewer believed that the topic was clearly and adequately discussed, and a score of “0” was given if a reviewer believed that a topic was not clearly and adequately discussed during the video. Colors are used to represent agreement (green or red) or disagreement (yellow) between the two reviewers.

The scorers' video evaluations using the NSC are displayed in Figure 1. Across all videos, the mean NSC score was 3.05 (±0.96), the modified DISCERN score was 13.45 (±3.42), and the DISCERN bias score was 3.36 (±0.79) (Table 2). ICCs between the two independent reviewers were excellent for both NSC (ICC = 0.997) and modified DISCERN (ICC = 0.949).^29^ There was a significant positive correlation between total NSC and modified DISCERN scores (*r* = .833, *p* < .01). Academic videos scored significantly higher on the NSC score than other videos (mean difference = 0.98, 95% CI: 0.16–1.80, *p* = .02). There was no significant difference between academic and other videos in the modified DISCERN or DISCERN bias scores (Table 5).

**TABLE 5:** Primary outcome results by video uploader type

Outcome	Overall	Academic	Other	*p*‐value
Novel Sialendoscopy Criteria Score	3.05 (±0.96)	3.71 (±0.81)	2.73 (±0.88)	.02*
Modified DISCERN Score	13.45 (±3.42)	15.14 (±2.01)	12.67 (±3.70)	.12
DISCERN Bias Score	3.36 (±0.79)	3.64 (±0.48)	3.23 (±0.88)	.27

*Note*: Assessments of video comprehensiveness and quality stratified by uploader type. Data are represented as means (±SD). Reported *p*‐value represents difference between academic and other videos. Asterisk (*) indicates significance (*p* < .05).

Abbreviation: SD, standard deviation.

AV quality score, reported video metrics, and calculated video metrics for all videos are displayed (Table 6). Academic videos had significantly higher AV quality scores than videos from other uploaders (mean difference = 0.73, 95% CI: 0.35–1.11, *p* = .01). Videos that were from other sources had significantly longer duration than those from academic medical institutions (276.3 ± 180 s vs. 156.7 ± 82 s, *p* < .05). There were no other significant differences in secondary outcomes between the two groups.

**TABLE 6:** Secondary outcome results by video uploader type

Metric	Overall	Academic	Other	*p*‐value
AV quality score (0–3)	2.43 (±0.64)	2.93 (±0.19)	2.20 (±0.65)	.009*
Views	18,208 (±30,023.13)	17,933 (±39,339.00)	18,337 (±26,213)	.977
Comments	23.59 (±52.94)	11.50 (±23.00)	27.31 (±59.50)	.617
Duration (seconds)	238.23 (±163.49)	156.71 (±81.70)	276.27 (±179.90)	.045*
Likes	67.89 (±135.87)	54.33 (±122.89)	74.15 (±145.82)	.777
Dislikes	8.79 (±14.49)	8.33 (±19.45)	9.00 (±12.54)	.929
Like ratio	88.15 (±17.45)	0.79 (±0.39)	0.59 (±0.44)	.434
View ratio	10.60 (±17.38)	7.47 (±12.28)	12.06 (±19.53)	.577
VPI	12.46 (±19.12)	7.42 (±12.47)	15.25 (±22.18)	.485

*Note*: Reported and calculated video metrics stratified by video uploader type. Data are represented as means (±SD). Reported *p*‐value represents difference between academic and other videos. Asterisk (*) indicates significance (*p* < .05).

Abbreviations: AV, audiovisual; SD, standard deviation; VPI, video power index.

Across all videos, AV score was positively correlated with NSC score (*R* = .429, *p* < .05) but not modified DISCERN score. Neither NSC nor modified DISCERN scores significantly correlated with any other secondary characteristics. Within secondary characteristics, AV score was significantly positively correlated with like ratio (*R* = .749, *p* < .01) and negatively correlated with days since upload (*R* = −.53, *p* = .01).

## 4 DISCUSSION

This is the first study to evaluate the quality and comprehensiveness of sialendoscopy information within YouTube videos for patients. Using the search term “sialendoscopy,” only 22 of the first 150 videos (14.7%) met inclusion criteria, highlighting the paucity of suitable sialendoscopy video content for patients. Users typically only click on videos found within the first search engine result page (SERP), with less than 1% of all user SERP clicks occurring beyond the first page and the likelihood of interaction significantly diminishing as search position increases.^16^, ^17^, ^20^ Even in the first 25 search results, only 8 (32%) videos were suitable for patients. Thus, YouTube may not be an appropriate resource for patient's to reliably find sialendoscopy videos suitable to their education level. Physicians may consider directing patients toward other resources, as most users are likely unwilling or unable to reliably search for an appropriate video within the large quantities of unsuitable videos on YouTube.

Given the paucity of videos for patients, it is important to consider who else may be using YouTube for information on sialendoscopy. One hundred‐nine (72.7%) of the initial 150 videos were excluded for being surgical technique videos/OR recordings or lectures for medical professionals that contained technical language or medical jargon. Of these excluded videos, 15 (60%) were in the top 25 search results. This suggests that physicians may be using YouTube to share sialendoscopy content with other physicians or medical personnel. Physicians frequently use online videos to study medical concepts: one study reported that 90% of polled physicians used online videos and, of these physicians, 86% specifically used YouTube.^30^ Future research may seek to investigate the quality and utility of physician‐oriented YouTube educational videos.

The overall low NSC and modified DISCERN scores indicate poor video comprehensiveness and quality, respectively. These findings are consistent with our initial hypothesis and reflect the findings of other surgery literature investigating the health information within YouTube videos.^14^, ^15^, ^19^, ^21^, ^22^ Within the overall low scores, academic videos scored significantly higher on NSC than videos uploaded from other sources, suggesting that academic uploaders provide patients with more comprehensive information. While otolaryngologists may encourage patients to seek videos uploaded from academic sources to access the most comprehensive information, YouTube overall appears to be a poor source of quality health information on sialendoscopy.

The videos included in this study were poor at discussing complications. None (0%) of the videos described intra‐procedural complications, and only three videos (13.7%) discussed any complications; these low reporting rates are consistent with our initial hypothesis and literature in other fields.^22^, ^23^ This lack of discussion may be due to the low complication rate of sialendoscopy procedures overall or because not all included videos were intended for patient education.^7^, ^31^ Physicians may choose to increase discussion of these topics in the future if their goals are to create comprehensive videos on sialendoscopy.

The above findings highlight that YouTube may not be an ideal resource for physicians to recommend to their patients. Physicians seeking to use YouTube for sialendoscopy patient education may consider preselecting videos at their own discretion and providing their patients with weblinks. This would eliminate the need for patients to siphon through the large quantity of videos intended for medical professionals and encourage targeted selection of higher quality videos. Physicians seeking to create their own high‐quality videos may use the NSC as a reference for pertinent inclusion points.

Viewers of online videos often perceive more popular videos as more credible sources of information.^32^ We found no correlations between the two scoring criteria (NSC and modified DISCERN) and popularity, indicating that more popular videos are not necessarily of higher quality. Moreover, although videos uploaded from academic sources were on average more comprehensive than from other sources, there was no difference in popularity between uploader sources. Taken together, this suggests that a highly informative video in and of itself will not ensure viewership, and it highlights an opportunity for academic medical institution uploaders to use strategies to increase video viewership, as even the best videos will not impact patient education if they cannot be viewed.

Growing bodies of literature studying online user behavior have outlined strategies to increase the likelihood of video viewership and can be implemented by otolaryngologists seeking to create content that is both highly informative and accessible. When a YouTube search is queried, the most popular videos appear at the top of YouTube's SERP.^20^ Strategies to maximize a video's SERP presence include (1) using thoughtful “keyword” and title selection, (2) sharing the video on corresponding social media platforms, and (3) keeping the video within the 3–5 min range.^20^ Keywords and titles should be in colloquial language and kept consistent throughout the video title, the text description, and within corresponding social media posts. The uploader may identify other popular sialendoscopy YouTube videos and choose identical keywords or similar title verbiage to mirror these videos' SERP successes.^20^, ^33^ Promoting the video on corresponding social media pages (i.e., Twitter, Instagram) will further establish video validity and direct users of multiple media networks back to YouTube.^20^, ^32^ Reducing video length augments engagement and increases the likelihood that the video will be watched to completion, a metric than has been shown to improve SERP.^20^, ^34^ While academic organizations can seek to implement these strategies themselves, various internet services exist to guide creators toward successful content creation and promotion, including the YouTube Creator Academy and Google Trends for YouTube.^20^, ^35^

Videos uploaded from academic medical institutions had significantly higher AV quality scores. While AV quality score did positively correlate with like ratio, it did not correlate with any other metrics of user engagement, such as number of views or VPI. While this suggests that academic medical institutions can produce high AV quality videos, the patients cannot appreciate such quality if it is not viewed. These findings reiterate the importance of physicians engaging in strategies to maximize their position on YouTube's SERP.

This study has potential limitations. Our small sample size may fail to represent the true extent of videos available to patients on sialendoscopy. However, as discussed previously, such a sample size is a finding itself that highlights the scarcity of videos available to patients on the topic. Furthermore, although we excluded videos that were not suitable for patients and found no difference in the DISCERN bias score between uploaders, we did not categorize videos by their intended purpose. Thus, it is possible that some of the remaining videos were not intended directly for patient education, potentially creating bias in certain discussion points based on the uploader's intentions. Future investigations should seek to evaluate video bias as it relates to the video's intended purpose (e.g., educational, testimonial, or marketing). Additionally, while this study provides valuable insight into the quality and comprehensiveness of sialendoscopy YouTube videos, it does not evaluate videos uploaded outside of YouTube and it does not reveal the extent to which these videos may truly educate patients. Finally, although the modified DISCERN has been extensively used to evaluate online patient education, no validation studies specific to the modified instrument have been published. Further studies may seek to validate the modified DISCERN score against other previously validated reliability grading systems.

## 5 CONCLUSION

Our study highlights the overall paucity and low quality of videos for patients on sialendoscopy. More popular videos are not necessarily higher quality or more comprehensive, and videos uploaded on YouTube are targeted more toward physicians rather than patients. As YouTube becomes an increasingly used source of health information, there is opportunity for otolaryngologists to produce more informative videos for patients while implementing targeted strategies to increase viewership.

## FUNDING INFORMATION

This research did not receive any specific grant from funding agencies in the public, commercial, or not‐for‐profit sectors.

## CONFLICT OF INTEREST

The authors have no funding, financial relationships, conflicts of interest, or competing interests to disclose.

## References

[1] Fox S . Health Topics. Pew Research Center; 2011. Accessed April 19, 2021. https://www.pewresearch.org/internet/2011/02/01/health-topics-2/

[2] Alexa . Alexa ‐ Top sites. Alexa: An AmazonCom Company; 2021. Accessed March 29, 2021. https://www.alexa.com/topsites

[3] National Cancer Institute . Health Information National Trends Survey (HINTS); 2021. Accessed April 21, 2021. https://hints.cancer.gov/Default.aspx

[4] Statista . Mobile apps: U.S. Smartphone Audience Reach; 2021. Accessed November 16, 2021. https://www.statista.com/statistics/281605/reach-of-leading-us-smartphone-apps/

[5] Sionis S , Caria RA , Trucas M , Brennan PA , Puxeddu R . Sialoendoscopy with and without holmium:YAG laser‐assisted lithotripsy in the management of obstructive sialadenitis of major salivary glands. Br J Oral Maxillofac Surg. 2014;52:58‐62. doi:10.1016/j.bjoms.2013.06.015 24280118

[6] Fabie JE , Kompelli AR , Naylor TM , Nguyen SA , Lentsch EJ , Gillespie MB . Gland‐preserving surgery for salivary stones and the utility of sialendoscopes. Head Neck. 2019;41:1320‐1327. doi:10.1002/hed.25560 30549387

[7] Galdermans M , Gemels B . Success rate and complications of sialendoscopy and sialolithotripsy in patients with parotid sialolithiasis: a systematic review. Oral Maxillofac Surg. 2020;24:145‐150. doi:10.1007/s10006-020-00834-x 32162129

[8] Atienza G , López‐Cedrún JL . Management of obstructive salivary disorders by sialendoscopy: a systematic review. Br J Oral Maxillofac Surg. 2015;53:507‐519. doi:10.1016/j.bjoms.2015.02.024 25823614

[9] Coniglio AJ , Deal AM , Bhate O , Hackman TG . In‐office versus operating room sialendoscopy: comparison of outcomes, patient time burden, and charge analysis. Otolaryngol Head Neck Surg. 2019;160:255‐260. doi:10.1177/0194599818813101 30453822

[10] Mastrolonardo E , Stewart M , Alapati R , et al. Improved efficiency of sialendoscopy procedures at an ambulatory surgery center. Am J Otolaryngol. 2021;42:102927. doi:10.1016/j.amjoto.2021.102927 33516124

[11] Weigelt F , Borzikowsky C , Hoffmann M , Laudien M . Success of minimally invasive salivary gland surgery‐quality of life, prognostic factors. Laryngoscope Investig Otolaryngol. 2020;5:832‐838. doi:10.1002/lio2.450 PMC7585237 33134529

[12] Vila PM , Olsen MA , Piccirillo JF , Ogden MA . Rates of sialoendoscopy and sialoadenectomy in 5,111 adults with private insurance. Laryngoscope. 2019;129:602‐606. doi:10.1002/lary.27243 30556133 PMC6379123

[13] Desai T , Shariff A , Dhingra V , Minhas D , Eure M , Kats M . Is content really king? An objective analysis of the public's response to medical videos on YouTube. PLoS One. 2013;8:e82469. doi:10.1371/journal.pone.0082469 24367517 PMC3867348

[14] Haymes AT , Harries V . “How to stop a nosebleed”: an assessment of the quality of epistaxis treatment advice on YouTube. J Laryngol Otol. 2016;130:749‐754. doi:10.1017/S0022215116008410 27345303

[15] Steinberg PL , Wason S , Stern JM , Deters L , Kowal B , Seigne J . YouTube as source of prostate cancer information. Urology. 2010;75:619‐622. doi:10.1016/j.urology.2008.07.059 19815255

[16] iProspect . iProspect search engine user behavior study. 2006. Accessed April 20, 2021. http://www.iprospect.com/.

[17] Dean B . We Analyzed 5 Million Google Search Results. Here's What We Learned About Organic CTR. Backlinko LLC; 2019.

[18] Enver N , Doruk C , Kara H , Gürol E , Incaz S , Mamadova U . YouTube™ as an information source for larynx cancer: a systematic review of video content. Eur Arch Otorhinolaryngol. 2020;277:2061‐2069. doi:10.1007/s00405-020-05906-y 32180014

[19] Aydin MA , Akyol H . Quality of information available on youtube videos pertaining to thyroid cancer. J Cancer Educ. 2020;35:599‐605. doi:10.1007/s13187-019-01502-9 30838529

[20] Haslam K , Doucette H , Hachey S , et al. YouTube videos as health decision aids for the public: an integrative review. Can J Dent Hyg. 2019;53:53‐66. 33240342 PMC7533808

[21] Wu V , Lee DJ , Vescan A , Lee JM . Evaluating youtube as a source of patient information for functional endoscopic sinus surgery. Ear Nose Throat J. 2020;101:401. doi:10.1177/0145561320962867 33021839

[22] Singh SK , Liu S , Capasso R , Kern RC , Gouveia CJ . YouTube as a source of information for obstructive sleep apnea. Am J Otolaryngol. 2018;39:378‐382. doi:10.1016/j.amjoto.2018.03.024 29605236

[23] Derakhshan A , Lee L , Bhama P , Barbarite E , Shaye D . Assessing the educational quality of “YouTube” videos for facelifts. Am J Otolaryngol. 2019;40:156‐159. doi:10.1016/j.amjoto.2019.01.001 30661892

[24] Charnock D , Shepperd S , Needham G , Gann R . DISCERN: an instrument for judging the quality of written consumer health information on treatment choices. J Epidemiol Community Health. 1999;53:105‐111. doi:10.1136/jech.53.2.105 10396471 PMC1756830

[25] Radonjic A , Fat Hing NN , Harlock J , Naji F . YouTube as a source of patient information for abdominal aortic aneurysms. J Vasc Surg. 2020;71:637‐644. doi:10.1016/j.jvs.2019.08.230 31611104

[26] Rees CE , Ford JE , Sheard CE . Evaluating the reliability of DISCERN: a tool for assessing the quality of written patient information on treatment choices. Patient Educ Couns. 2002;47:273‐275. doi:10.1016/s0738-3991(01)00225-7 12088606

[27] Ocak U . Evaluation of the content, quality, reliability and accuracy of YouTube videos regarding endotracheal intubation techniques. Niger J Clin Pract. 2018;21:1651‐1655. doi:10.4103/njcp.njcp_207_18 30560831

[28] Celik H , Polat O , Ozcan C , Camur S , Kilinc BE , Uzun M . Assessment of the quality and reliability of the information on rotator cuff repair on youtube. Orthop Traumatol Surg Res. 2020;106:31‐34. doi:10.1016/j.otsr.2019.10.004 31882329

[29] Koo TK , Li MY . A guideline of selecting and reporting intraclass correlation coefficients for reliability research. J Chiropr Med. 2016;15:155‐163. doi:10.1016/j.jcm.2016.02.012 27330520 PMC4913118

[30] Rapp AK , Healy MG , Charlton ME , Keith JN , Rosenbaum ME , Kapadia MR . Youtube is the most frequently used educational video source for surgical preparation. J Surg Educ. 2016;73:1072‐1076. doi:10.1016/j.jsurg.2016.04.024 27316383 PMC7263439

[31] Maresh A , Kutler DI , Kacker A . Sialoendoscopy in the diagnosis and management of obstructive sialadenitis. Laryngoscope. 2011;121:495‐500. doi:10.1002/lary.21378 21298637

[32] Michalovich A , Hershkovitz A . Assessing YouTube science news' credibility: the impact of web‐search on the role of video, source, and user attributes. Public Underst Sci. 2020;29:376‐391. doi:10.1177/0963662520905466 32072863

[33] Zhou R , Khemmarat S , Gao L , et al. Boosting video popularity through keyword suggestion and recommendation systems. Neurocomputing. 2016;205:529‐541. doi:10.1016/j.neucom.2016.05.002

[34] Welbourne DJ , Grant WJ . Science communication on YouTube: factors that affect channel and video popularity. Public Underst Sci. 2016;25:706‐718. doi:10.1177/0963662515572068 25698225

[35] Chelaru S , Orellana‐Rodriguez C , Altingovde IS . How useful is social feedback for learning to rank YouTube videos? World Wide Web. 2014;17:997‐1025. doi:10.1007/s11280-013-0258-9

